# Supplementary material for: Single-phase perovskite oxide with super-exchange induced atomic-scale synergistic active centers enables ultrafast hydrogen evolution
Source: Nat Commun. 2020 Nov 9;11:5657. doi: 10.1038/s41467-020-19433-1 (PMC7653924; doi:10.1038/s41467-020-19433-1)
Supplement: Supplementary file 1 — Supplementary information [file 41467_2020_19433_MOESM1_ESM.pdf]

Single-phase perovskite oxide with super-exchange induced atomic-scale synergistic active centers enables ultrafast hydrogen evolution

Dai *et al.*

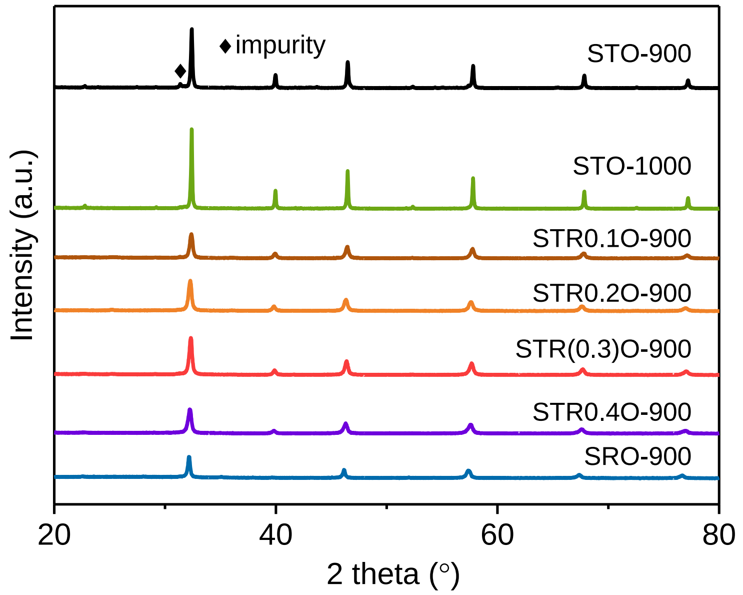

Supplementary Figure 1. XRD patterns of $SrTi_{1-x}Ru_xO_{3-\delta}$, (x=0, 0.1, 0.2, 0.3, 0.4, 1) prepared at calcination temperatures of 900 and 1000 °C. $SrTiO_3$, $SrTi_{0.9}Ru_{0.1}O_{3-\delta}$, $SrTi_{0.8}Ru_{0.2}O_{3-\delta}$, $SrTi_{0.7}Ru_{0.3}O_{3-\delta}$, $SrTi_{0.6}Ru_{0.4}O_{3-\delta}$, $SrRuO_3$ are denoted as STO, STR0.1O, STR0.2O, STR(0.3)O, STR0.4O and SRO, respectively. In addition to the standard cubic phase in XRD patterns of STO-900, some impurity phases were also observed. To avoid complications from the effect of impurity phases on electrocatalytic activity, the calcination temperature for the formation of pure STO phase was chosen to be 1000 °C for study in this work.

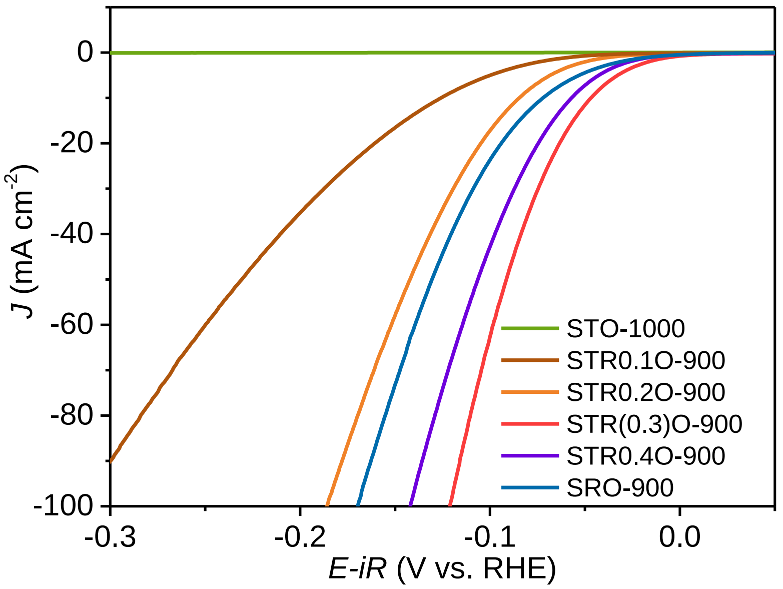

Supplementary Figure 2. Polarization curves of $SrTi_{1-x}Ru_xO_{3-\delta}$ (x=0, 0.1, 0.2, 0.3, 0.4, 1) in Ar-saturated 1 M KOH solution at a scan rate of 5 mV s^{-1}.

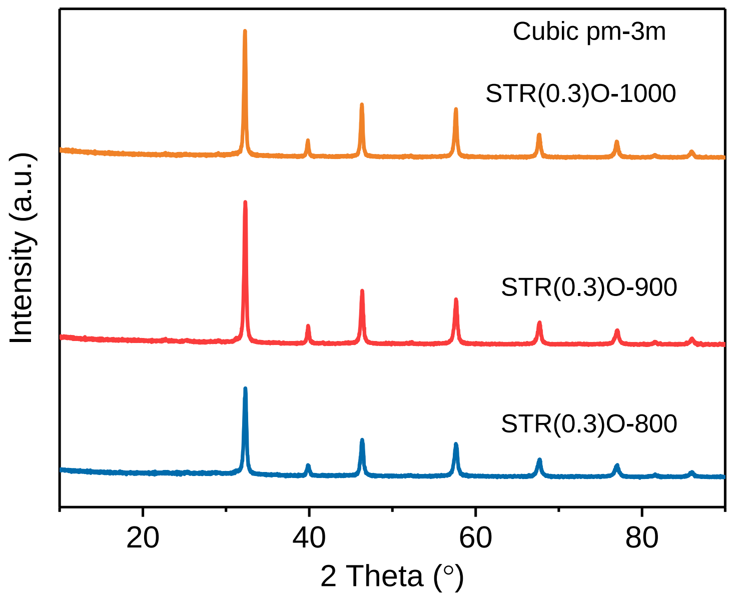

Supplementary Figure 3. XRD patterns of STRO prepared at different calcination temperatures of 800, 900, and 1000 °C.

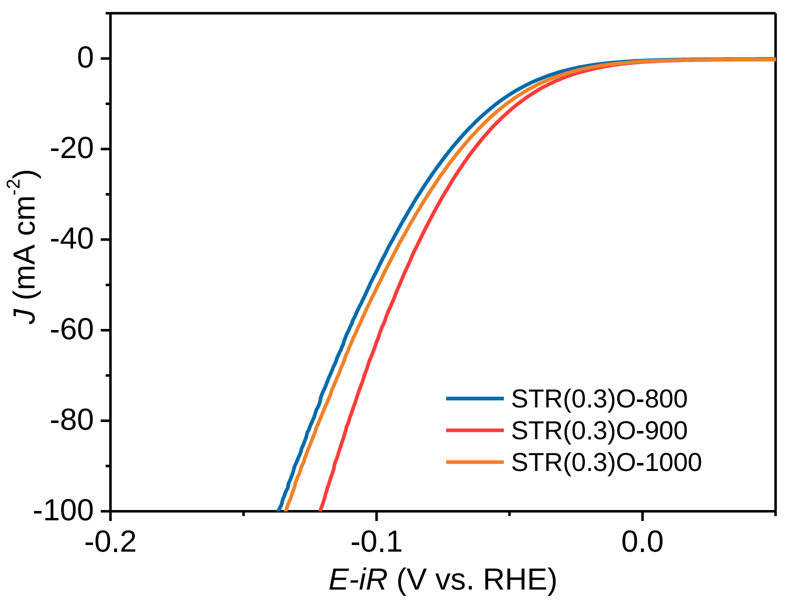

Supplementary Figure 4. Polarization curves of STRO samples at different calcination temperatures in Ar-saturated 1 M KOH solution at a scan rate of 5 mV s^{-1}.

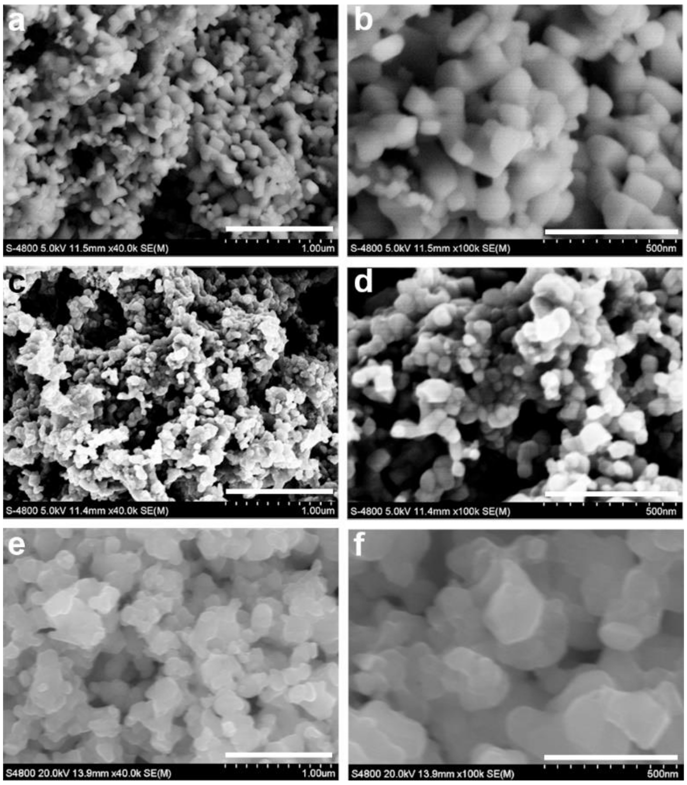

Supplementary Figure 5. SEM images with different magnifications. (**a**,**b**) STO, (**c**,**d**) STRO, and (**e**,**f**) SRO samples. Scale bar in (**a**,**c**,**e**) is 1 μm, in (**b**,**d**,**f**) is 500 nm.

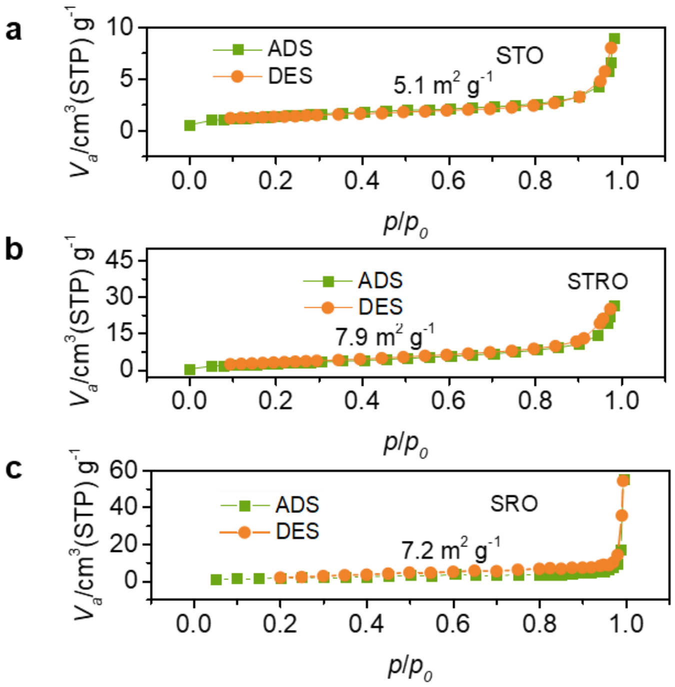

Supplementary Figure 6. N_2 adsorption-desorption isotherm. (**a**) STO, (**b**) STRO and (**c**) SRO and the corresponding specific surface areas as estimated from Brunauer-Emmett-Teller (BET) measurements.

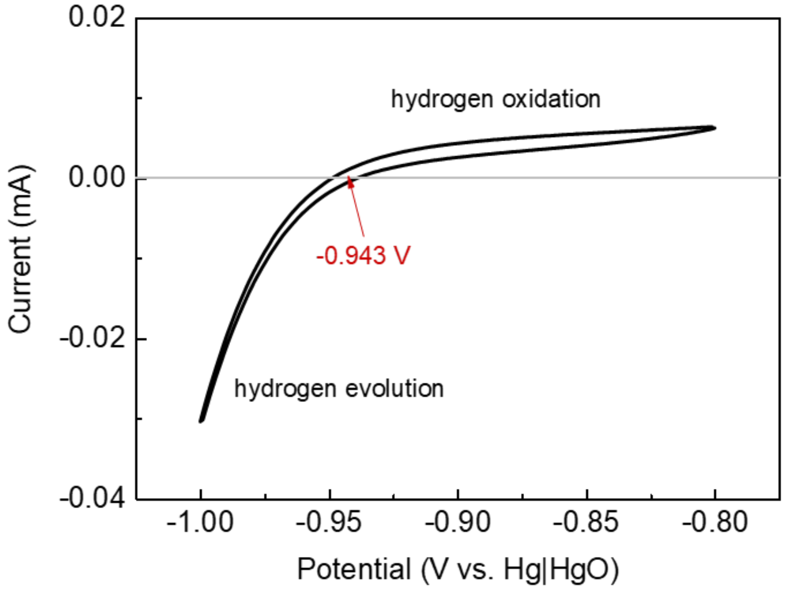

Supplementary Figure 7. RHE calibration of the Hg|HgO reference electrode in 1 M KOH. The calibration process was performed in a high purity H_2-saturated 1 M KOH with a platinum RDE (PINE, 0.126 cm^2) as the working electrode, Pt foil as the counter electrode, and Hg|HgO (1 M KOH) as the reference electrode. Cyclic voltammetry (CV) was conducted at a scan rate of 1 mV s^{-1}, and the average of the two potentials at which the current crossed zero was taken as the thermodynamic potential for the hydrogen electrode reaction. In 1 M KOH solution, $E_{RHE} = E_{Hg|HgO}$ +0.943 V.

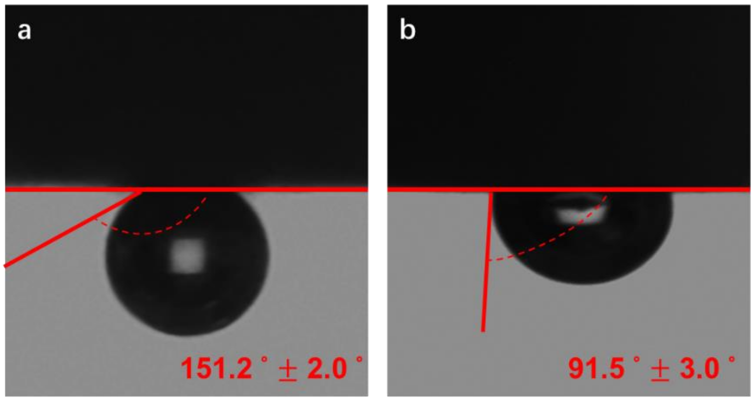

Supplementary Figure 8. The contact angle of hydrogen bubbles in 1 M KOH solution. (**a**) STRO and (**b**) Pt/C.

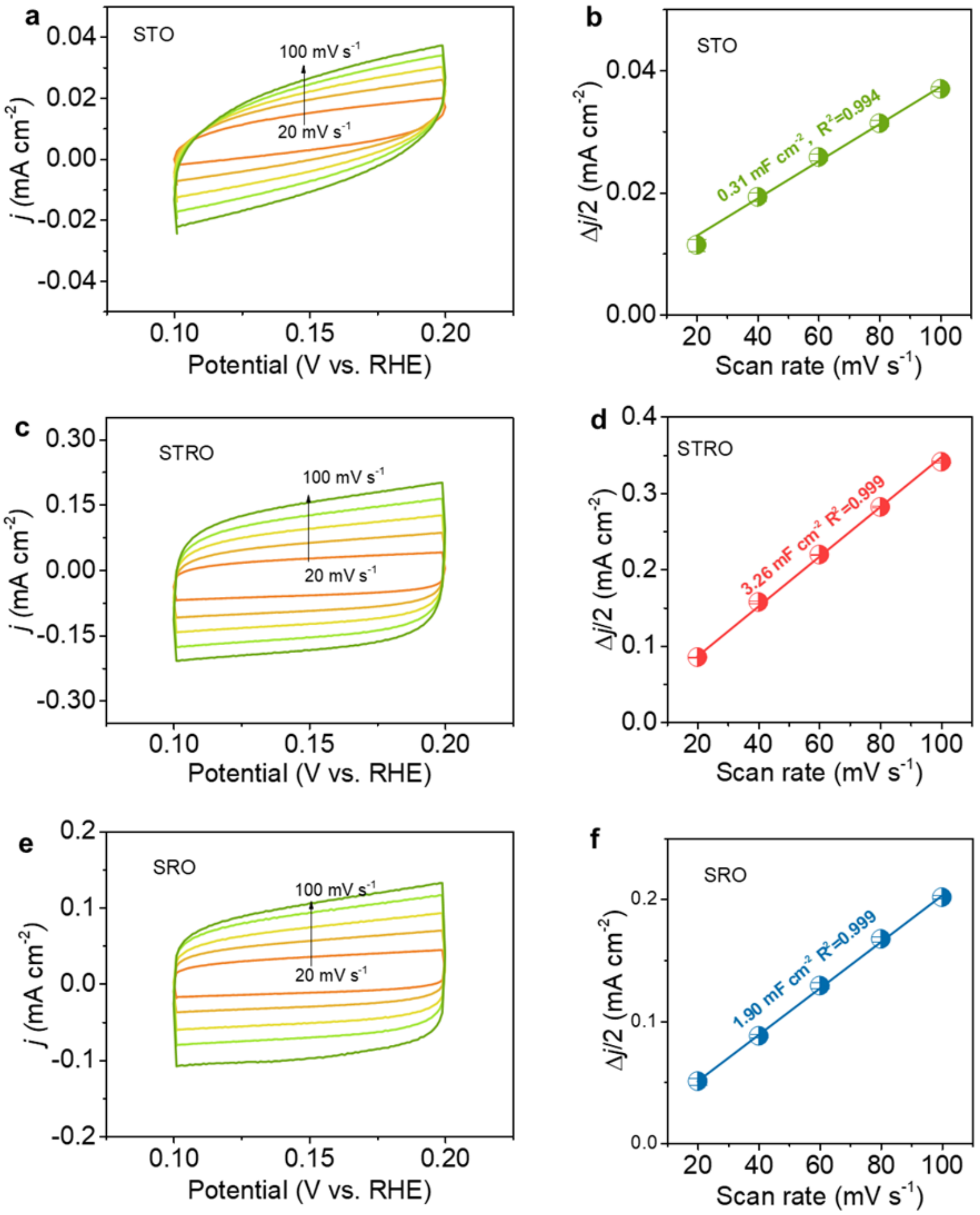

Supplementary Figure 9. ECSA estimation determined from C_{dl}. The C_{dl} obtained from a cyclic voltammetry (CV) method is expected to be linearly proportional to the ECSA[1-3]. CV measurements in a non-faradic current region (0.1-0.2 V vs. RHE, no *iR*-corrected) at scan rates of 20, 40, 60, 80 and 100 mV s^{-1} of (**a**) STO, (**c**) STRO and (**e**) SRO catalysts in 1 M KOH. Linear fitting of the capacitive currents versus CV scan rates for (**b**) STO, (**d**) STRO and (**f**) SRO.

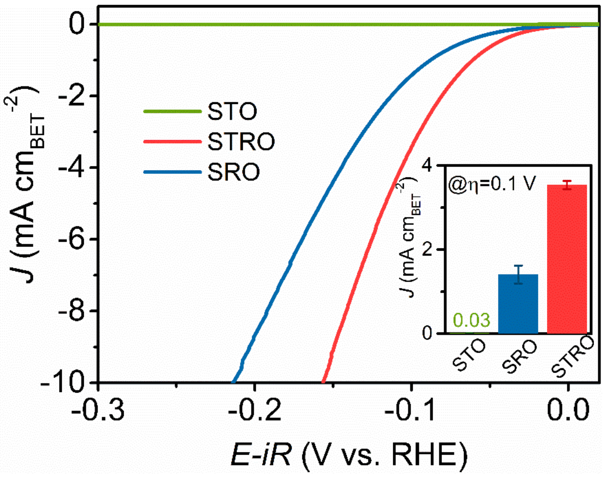

Supplementary Figure 10. Specific activity normalized to the real oxide surface area as a function of potential.

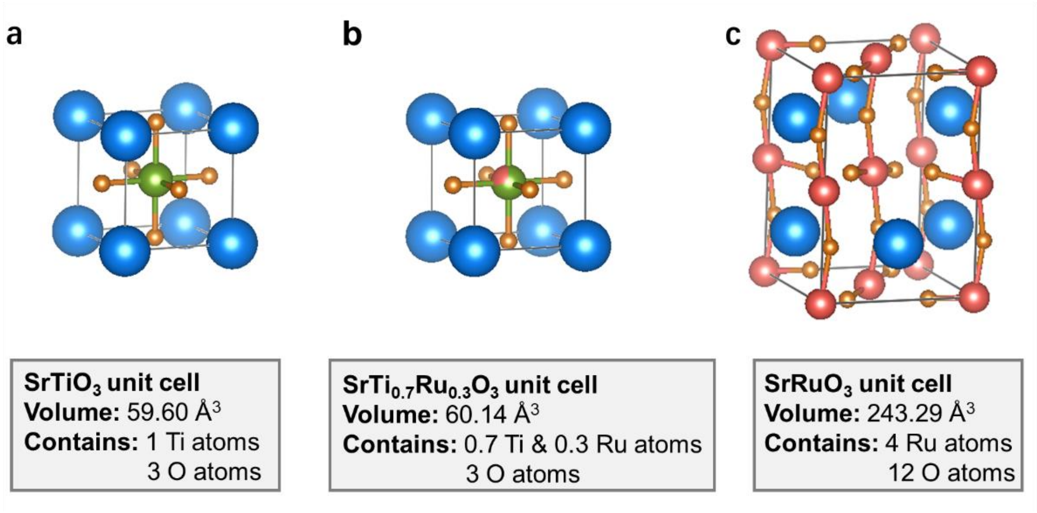

Supplementary Figure 11. Unit cell of catalysts. (**a**) STO, (**b**) STRO and (**c**) SRO unit cell. Detailed TOF calculations are given in Supplementary Note 1.

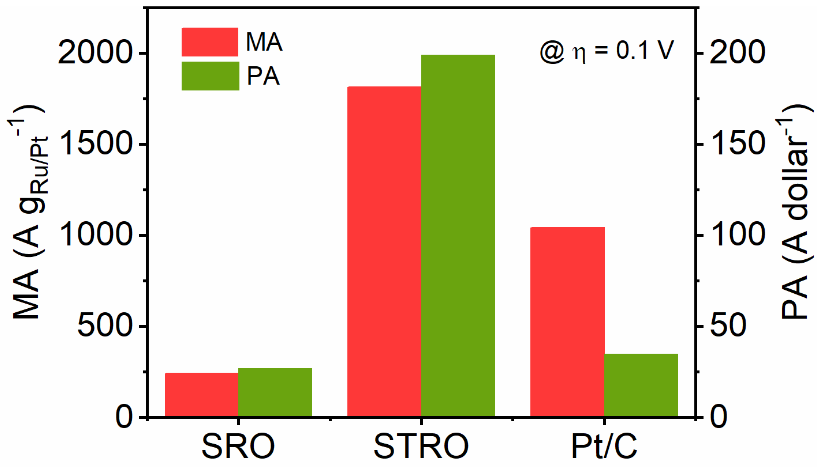

Supplementary Figure 12. Mass activity (MA) and price activity (PA) of SRO, STRO, and Pt/C at the overpotential of $\eta = 0.1$ V.

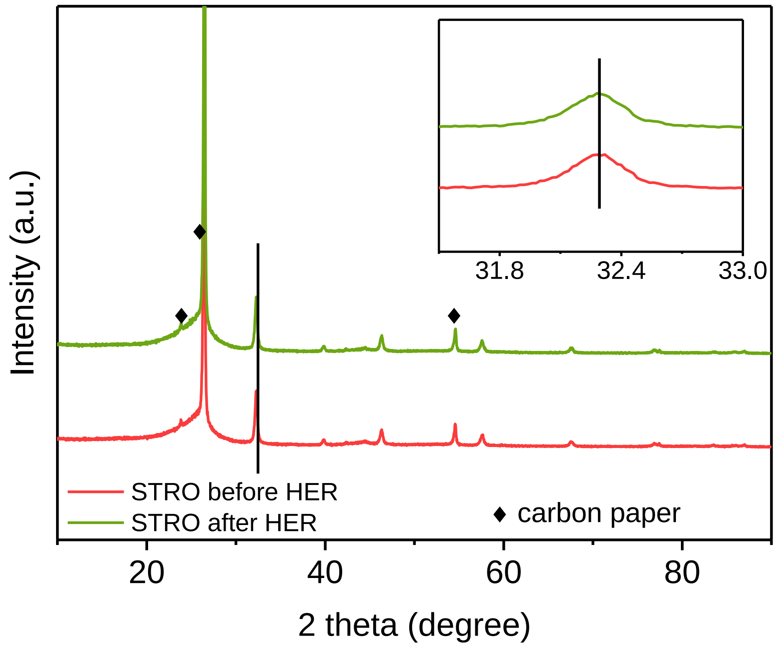

Supplementary Figure 13. XRD patterns of STRO before and after HER. The XRD patterns show that there is no apparent variation in the peak pattern and position of STRO before and after HER, confirming that the crystal structure of STRO does not change during HER.

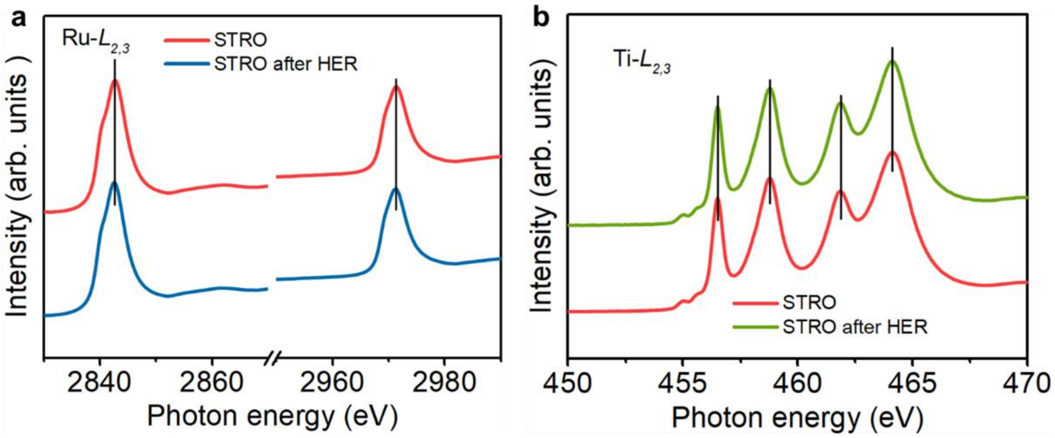

Supplementary Figure 14. XAS spectra. (**a**) Ru-$L_{2,3}$ and (**b**) Ti-$L_{2,3}$ for STRO before and after HER. The Ru-$L_{2,3}$ and Ti-$L_{2,3}$ spectra of STRO before and after HER show that there is no apparent variation, indicative of no electronic structure variation. The unchanged electronic structure therefore excludes the possibility of surface structure change during HER.

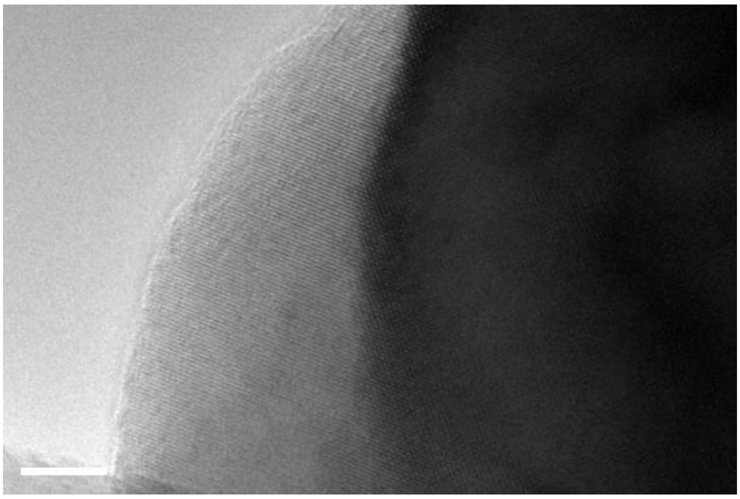

Supplementary Figure 15. TEM image of STRO after HER. Neither surface amorphization nor lattice change was observed, demonstrating the stability of crystal structure of STRO during HER. The scale bar is 5 nm.

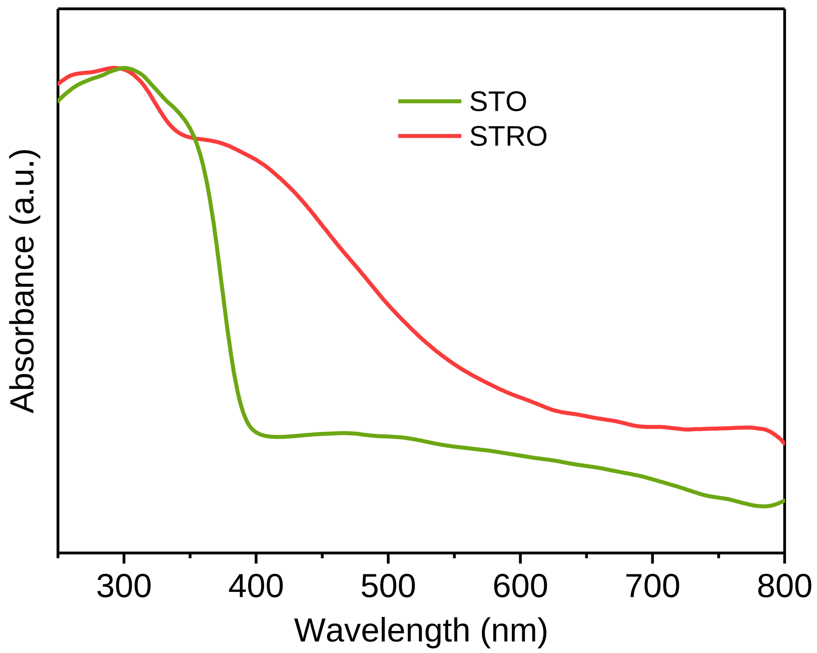

Supplementary Figure 16. The UV-is spectroscopy of STO and STRO.

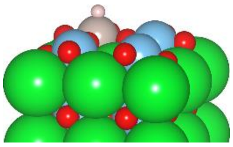	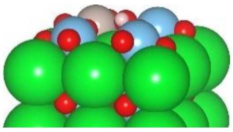	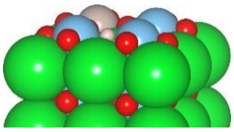
H@Ru<br>$\Delta G_{H^*} = -0.29\ eV$	H@O<br>$\Delta G_{H^*} = 0.06\ eV$	H@O vancancy<br>$\Delta G_{H^*} =\ 0.05\ eV$

Supplementary Figure 17. Surface structural models and calculated sites of STRO for H* adsorption.

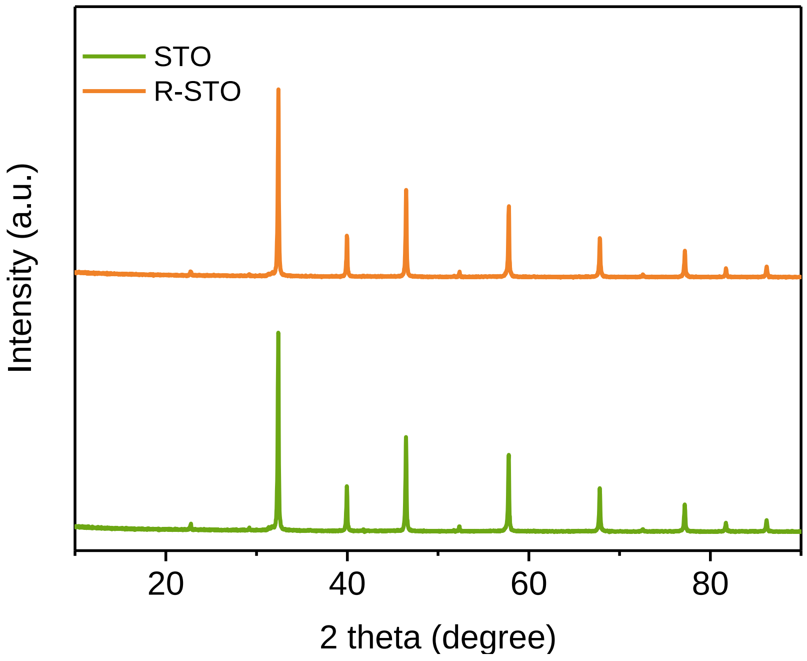

Supplementary Figure 18. XRD patterns of STO and R-STO. The R-STO was prepared by treating STO in a reductive atmosphere of 10% H_2/90% Ar at 500 °C for 2h. The main peak of R-STO slightly shifts to a lower angle compared to the STO, implying the reduction of Ti oxidation state and generation of oxygen vacancies.

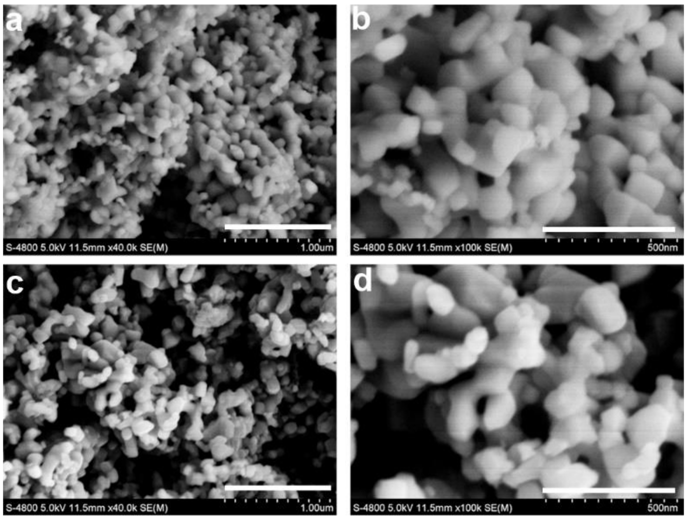

Supplementary Figure 19. SEM images with different magnifications. (**a**,**b**) STO and (**c**,**d**) R-STO samples. Scale bar in (**a**,**c**) is 1 μm, in (**b**,**d**) is 500 nm.

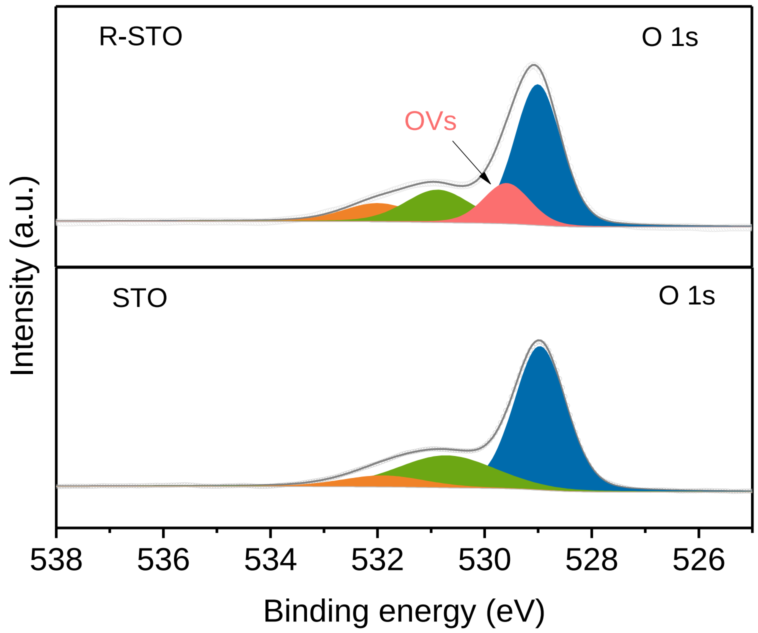

Supplementary Figure 20. The XPS spectra of O 1s for STO and R-STO. Much larger number of (O_2^{2-}/O^-) species was observed for STO relative to R-STO, implying the significantly increased oxygen vacancies.

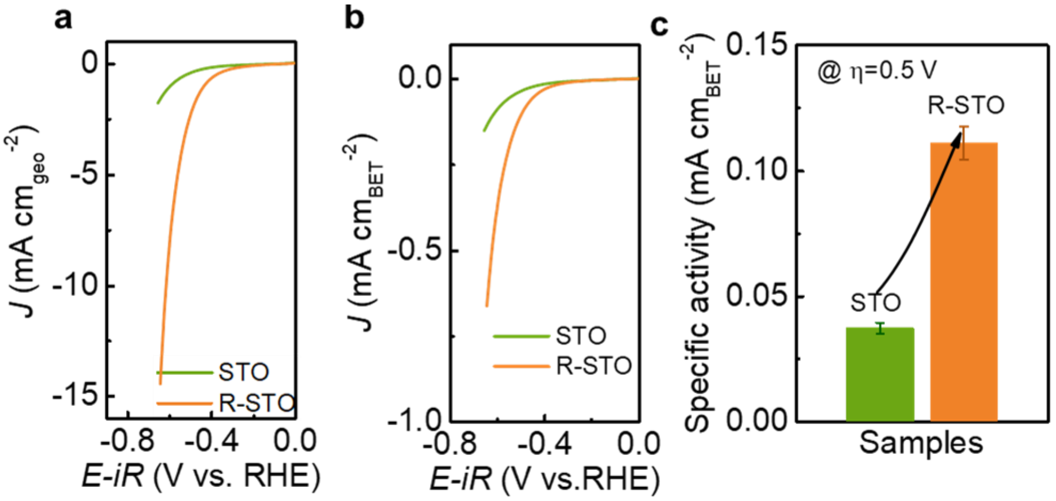

Supplementary Figure 21. HER activities. (**a**) HER electrode activity and (**b**,**c**) specific activity of STO and R-STO.

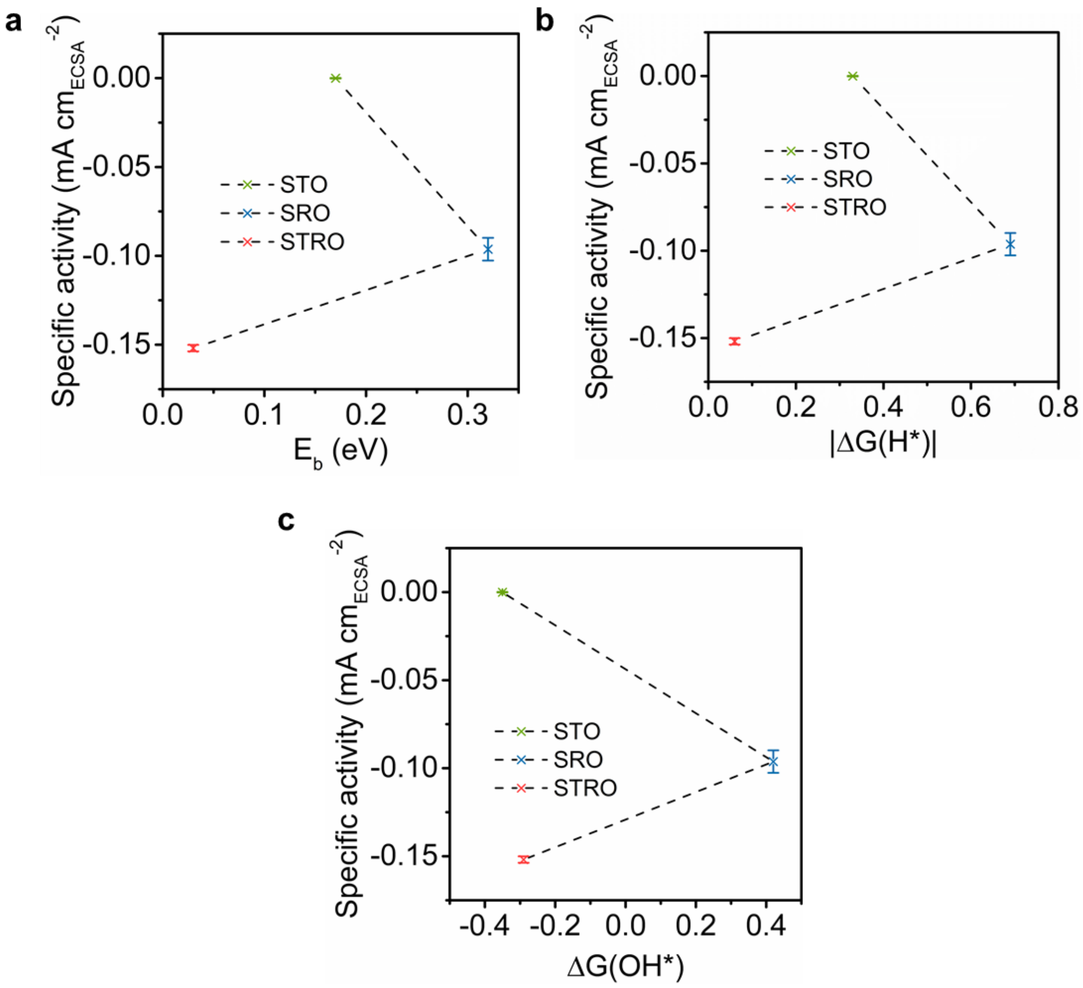

Supplementary Figure 22. Parameter-activity correlation. The relationship of (**a**) the kinetic reaction barriers (E_b), (**b**) the absolute binding energy for hydrogen(|ΔG_{H*}|), and (**c**) the hydroxide desorption energetics (ΔG_{OH*}) with the specific activity of STO, STRO and SRO.

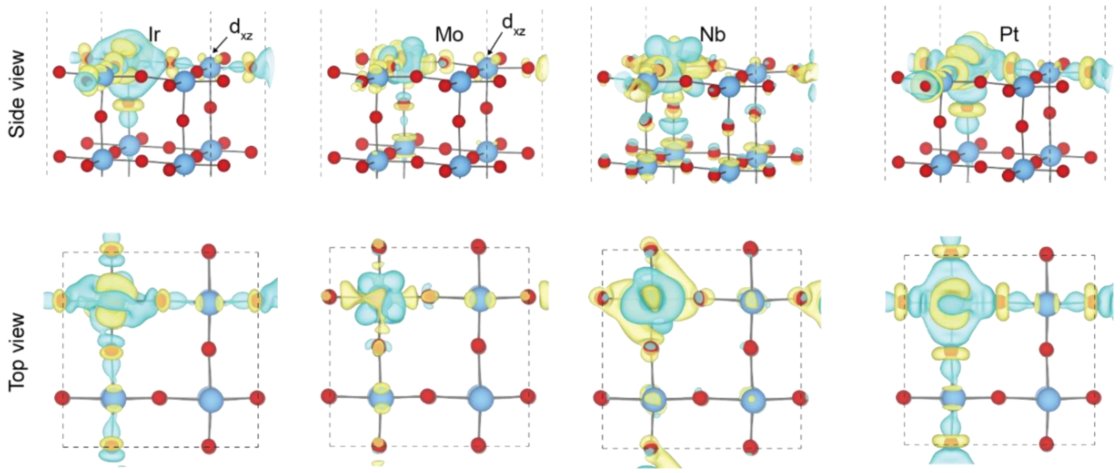

Supplementary Figure 23. Charge density redistribution upon the introduction of different metal dopants. From left to right: Ir, Mo, Nb and Pt.

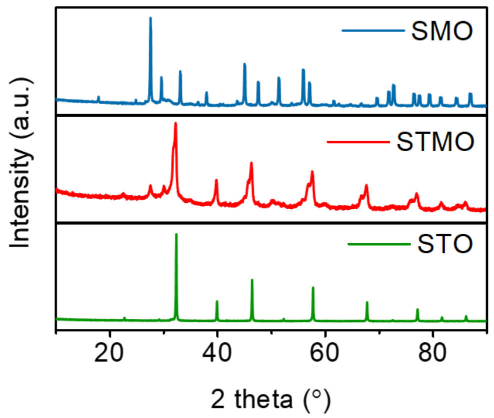

Supplementary Figure 24. XRD patterns of STO, $SrTi_{0.7}Mo_{0.3}O_{3-\delta}$ (STMO), and $SrMoO_4$ (SMO). Two weak peaks before the main peak of STMO could be indexed to the SMO.

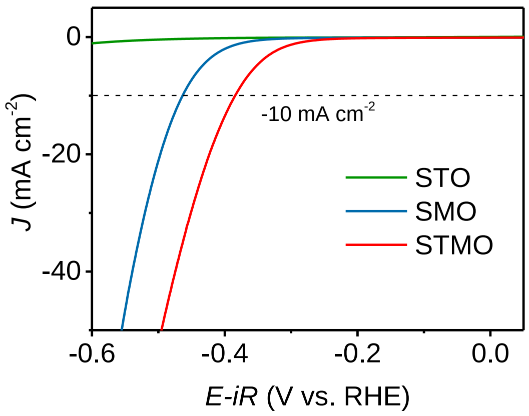

Supplementary Figure 25. Polarization curves of STO, STMO, and SMO catalysts in an Ar-saturated 1 M KOH solution. Scan rate, 5 mV s^{-1}.

Supplementary Table 1. The refined lattice parameters and reliability factors of STO, STRO and SRO samples.

Sample	Phase type	Space group	Lattice parameters						R_p(%)	R_{wp}(%)	GOF
			a (Å)	b (Å)	c (Å)	α(º)	β(º)	γ(º)			
STO	Cubic	*Pm-3m*	3.9061	3.9061	3.9061	90	90	90	7.47	9.70	1.64
STRO	Cubic	*Pm-3m*	3.9178	3.9178	3.9178	90	90	90	8.55	11.12	1.60
SRO	Orthorhombic	*Pnma*	5.5610	7.8674	5.5606	90	90	90	12.19	14.85	2.07

Supplementary Table 2. Summary of HER activity in alkaline media (1 M KOH) for various representative state-of-the-art catalysts, including metal oxides, synergistic hybrids, metal/alloy, single atoms, metal phosphides, metal sulfides, metal selenides, metal carbides, metal nitrides and functional carbon, etc.

Catalysts	Substrate	Mass loading (mg·cm^{-2})	η @10 mA cm^{-2} (mV)	Tafel slope (mV dec^{-1})	References
STRO	Glass carbon	0.232	46	40	This work
Metal oxides					
Sr_2RuO_4	Glass carbon	0.232	61	51	Ref [4]
Gd0.5	Glass carbon	0.232	210	29	Ref [5]
S-CoO NR	Carbon fiber paper	0.486	73	82	Ref [6]
2-Cylce $NiFeO_x$	Carbon fiber paper	1.6	88	150	Ref [7]
LBSCOF	Glass carbon	0.157	~180	44	Ref [8]
R-NCO	Ni foam	2.5	135	52	Ref [9]
Co_3O_4-MTA	Ni foam	N.A.[a]	~150	98	Ref [10]
$NiCo_2O_4$	Ni foam	~1	~100	50	Ref [11]
3D CoFeZr NS	Ni foam	N.A.	104	119	Ref [12]
Ni/Zn doped CoO NR	Carbon fiber paper	0.486	53	47	Ref [13]
SCFP	Ni foam	0.034	110	94	Ref [14]
Pr0.5BSCF	Glass carbon	0.232	237	45	Ref [15]
P-Co_3O_4	Ti mesh	0.4	120	52	Ref [16]
SNCF-NR	Glass carbon	0.232	232	103	Ref [3]
CuCoO	Ni foam	1.2	140	108	Ref [17]
NiO NRs-m-Ov	Carbon fiber paper	0.2	110	100	Ref [18]
Synergistic hybrids					
H_2-CoCat	FTO	N.A.	>385	140	Ref [19]
LSC&$MoSe_2$	Glass carbon	0.385	~250	34	Ref [20]
NiO@1T-MoS_2	Carbon fiber paper	1.02	46	52	Ref [21]
PtNi/NiS NW	Glass carbon	N.A.	42	N.A.	Ref [22]

Pt NW/SL -$Ni(OH)_2$	Glass carbon	N.A.	~70	N.A.	Ref [23]
Ni/NiO-CNT	Glass carbon	0.28	~80	82	Ref [24]
MoS_2/NiCo-LDH	Carbon fiber paper	N.A.	78	77	Ref [25]
Li_xNiO/Ni	Glass carbon	0.6	36	50	Ref [26]
NiO/PtNi	Glass carbon	~0.06	40	79	Ref [27]
MoP@NCHS	Glass carbon	0.4	92	62	Ref [28]
sc-$Ni_2P^{\sigma-}$/NiHO	Ni foam	N.A.	60	75	Ref [29]
Mo_3P/Mo NB	Glass carbon	~1.01	78	56	Ref [30]
TiO_2 ND/Co NSNT	Carbon fibers	0.75	108	62	Ref [31]
Ni_5P_4-Ru	Glass carbon	0.142	54	52	Ref [32]
$MoS_2/Ni(OH)_2$	Glass carbon	0.285	185	73	Ref [33]
$Co_{0.85}Se$/NiFe-LDH	Exfoliated graphene (EG) foil	4	260	160	Ref [34]
Co/Co_3O_4 NS	Ni foam	0.85	95	44	Ref [35]
NiO@Ni/WS_2	Carbon cloth	N.A.	40	83	Ref [36]
Ni_3N/Pt	Ni mesh	2	50	37	Ref [37]
MoS_2@NiO	Glass carbon	0.68	406	43	Ref [38]
nPBA@ $Co(OH)_2$	Ni foam	3.1	258	46	Ref [39]
NiFe/$NiCo_2O_4$	Ni foam	N.A.	105	88	Ref [40]
$NiCo_2O_4$/CuS	Carbon fiber paper	0.3	72	41	Ref [41]
MnO/hcp Ni	Glass carbon	2	80	68	Ref [42]
2D MoS_2@ $Co(OH)_2$	Glass carbon	0.2	89	53	Ref [43]
β-$Ni(OH)_2$/Pt	Glass carbon	N.A	115	42	Ref [44]
$MoSe_2/SnS_2$	Glass carbon	0.2	285	109	Ref [45]

$Ni(OH)_2$@CuS	Glass carbon	0.286	95	104	Ref [46]
$Ni(OH)_2/MoS_2$	Carbon cloth	N.A.	80	60	Ref [47]
Pt-$Co(OH)_2$	Carbon cloth	6.9	32	70	Ref [48]
Metals/alloys					
Au-Ru-2 NW	Glass carbon	0.08	50	31	Ref [49]
Ni-Mo-N	Carbon fiber cloth	2.9	40	70	Ref [50]
Ni-BDT-A	Carbon cloth	1	80	70	Ref [51]
Ni-N0.19	Carbon fiber paper	N.A.	42	125	Ref [52]
$CoSn_2$	Ni foam	N.A.	103	78	Ref [53]
Pt_3Ni_3 NW	Glass carbon	N.A.	50	N.A.	Ref [54]
NiCu@C	Graphite plate	0.384	74	94	Ref [55]
Ni_3Fe@N-C NT/NF	Glass carbon	~0.57	72	96	Ref [56]
Cu-Ni-Al	Glass carbon	N.A.	139	110	Ref [57]
Ni42-300	N.A.	N.A.	299	~117	Ref [58]
Co@NG	Glass carbon	0.47	220	112	Ref [59]
MnNi	Glass carbon	0.28	360	N.A.	Ref [60]
NiMo NW	Ni foam	0.41	270	86	Ref [61]
CuNi NC	Glass carbon	N.A.	140	79	Ref [62]
Single atoms					
Co_1/PCN	Glass carbon	0.5	89	59	Ref [63]
Pt@PCM	Glass carbon	N.A.	~150	74	Ref [64]
Pt_1/N-C	Glass carbon	0.25	46	37	Ref [65]
Pt/np-$Co_{0.85}$Se	Glass carbon	2.04	58	39	Ref [66]
Co_1N_x/C	Glass carbon	2	247	75	Ref [67]

Co_1/NG	Glass carbon	0.285	270	N.A.	Ref [68]
Mo_1/N_1C_2	Glass carbon	0.408	132	90	Ref [69]
Metal phosphides					
Holey NiCoP NS	Ni foam	1	58	57	Ref [70]
CoP/NCNHP	Glass carbon	~0.39	115	66	Ref [71]
CoP	Carbon cloth	0.92	209	129	Ref [72]
RuP_2@NPC	Glass carbon	1	52	69	Ref [73]
Ni_5P_4	Ni foil	25.8	150	53	Ref [74]
Fe-CoP	Ti foil	1.03	78	75	Ref [75]
CoMoP@C	Glass carbon	0.354	81	56	Ref [76]
np-$(Co_{0.52}Fe_{0.48})_2P$	N.A.	2.5	79	40	Ref [77]
MoP	Glass carbon	0.86	~150	48	Ref [78]
MoP@C	Carbon cloth	6	49	54	Ref [79]
$Zn_xCo_{1-x}P$	Titanium mesh	1.52	67	N.A.	Ref [80]
. $Cu_{0.3}Co_{2.7}P$/NC	Glass carbon	0.4	220	122	Ref [81]
MoP/CNT	Carbon fiber paper	0.5	86	N.A.	Ref [82]
NiCoP/rGO	Carbon fiber paper	0.15	209	124	Ref [83]
Ni-P	Carbon paper	N.A.	100	~85	Ref [84]
CoP-MNA	Ni foam	6.2	54	51	Ref [85]
Ce-doped CoP NW	Ti plate	0.2	92	64	Ref [86]
Ni_2P-Cu_3P	Porous NiCuC	N.A.	78	173	Ref [87]
Metal sulfides					
C-MoS_2	Carbon cloth	N.A.	45	46	Ref [88]
Cu ND/Ni_3S_2 NT	Carbon fibers	0.52	128	76	Ref [89]

$Zn_{0.30}Co_{2.70}S_4$	Glass carbon	0.285	85	N.A.	Ref [90]
Ni_3S_2	Ni foam	1.6	223	N.A.	Ref [91]
MoS_2/Ni_3S_2	Ni foam	9.7	110	83	Ref [92]
Ni-doped MoS_2	N.A.	0.89	98	60	Ref [93]
N-doped Ni_3S_2 NS	Ni foam	0.59	155	133	Ref [94]
$Ni_{0.33}Co_{0.67}S_2$ NW	Ti foil	0.3	88	118	Ref [95]
C, N-doped $NiPS_3$	Glass carbon	0.408	53	38	Ref [96]
$NiCo_2S_4$	Ni foam	N.A.	80	59	Ref [97]
$NiCo_2S_4$ NW	Ni foam	N.A.	210	59	Ref [98]
Co-O-1T-MoS_2/SWNT	Glass carbon	N.A	113	50	Ref [99]
V-doped NiS_2 NS	Glass carbon	0.272	110	90	Ref [100]
a-Ni_3S_2@NPC	Cu film	N.A.	61	68	Ref [101]
Metal selenides					
o-$CoSe_2$|P	Glass carbon	1.02	104	69	Ref [102]
Li-$IrSe_2$	Carbon fiber paper	0.25	72	N.A.	Ref [103]
Tubular $CoSe_2$ NS	Ni foam	N.A.	79	84	Ref [104]
NiSe	Ni foam	2.8	96	120	Ref [105]
$Ni_{0.89}Co_{0.11}Se_2$ MNSN	Ni foam	2.62	85	52	Ref [106]
c-$CoSe_2$	Carbon cloth	0.5	200	85	Ref [107]
SWCNT/MoSe	Glass carbon	2	63	63	Ref [108]
Metal carbides					
MoC_x NO	Glass carbon	0.8	151	59	Ref [109]
Mo_2C	Carbon paste	0.8	~190	54	Ref [110]
Fe_3C@NCNT	N.A.	N.A.	154	N.A.	Ref [111]
N@$MoPC_x$ NS	Glass carbon	0.14	139	87	Ref [112]
Mo_2C/N-doped C	Glass carbon	0.28	100	65	Ref [113]

N,P-doped Mo_2C@carbon NP	Glass carbon	0.9	50	71	Ref [114]
Mo_2C/CLCN	Glass carbon	0.357	~200	55	Ref [109]
Metal nitrides					
CoN_x/C	Glass carbon	2	170	75	Ref [68]
NiCoN/C	Glass carbon	0.2	130	N.A.	Ref [116]
Co-Ni_3N	Carbon cloth	2.91	194	156	Ref [117]
NiMoN	Carbon cloth	~1.1	109	95	Ref [118]
Ni_3N@CQD	Glass carbon	0.18	69	108	Ref [119]
Functional carbon materials					
C_3N_4@NG	Glass carbon	0.1	>600	N.A.	Ref [120]
ONPPGC	Carbon cloth	0.1	446	154	Ref [121]
N, S-CN	Glass carbon	N.A.	380	103	Ref [122]

[a]: N. A.=Not available

Supplementary Table 3. Results of Bader charge analysis.

Samples	Atoms	Charges (|e|)
STO	Ti	+2.2
SRO	Ru	+1.51
STRO	Ti	+2.1
	Ru	+1.84

Supplementary Table 4. O 1s XPS peak deconvolution results of STO and STRO.

Electrocatalysts	Lattice O^{2-}	O_2^{2-}/O^-	-OH or O_2	H_2O or CO_3^{2-}
STO	64.56%	/	27.39%	8.05%
STRO	41.07%	19.17%	31.37%	8.39%

Supplementary Table 5. Evaluation of STO, STRO and SRO catalysts based on four main key factors affecting the alkaline HER activity.

<table>
<tr><th colspan="2"></th><th>Electronic conductivity</th><th>Water dissociation</th><th>OH desorption</th><th>H adsorption</th></tr>
<tr><td colspan="2">STO</td><td>×</td><td>√</td><td>×</td><td>×</td></tr>
<tr><td colspan="2">SRO</td><td>√</td><td>×</td><td>√</td><td>×</td></tr>
<tr><td rowspan="3">STRO</td><td>Ti site</td><td>√</td><td>√</td><td>×</td><td>√</td></tr>
<tr><td>Ru site</td><td>√</td><td>×</td><td>√</td><td>√</td></tr>
<tr><td>Ti+Ru site</td><td>√</td><td>√ (on Ti)</td><td>√ (on Ru)</td><td>√</td></tr>
</table>

Note: √ means favorable; × means unfavorable.

Supplementary Note 1. Turnover frequency calculations

To calculate the per-site turnover frequency (TOF), we used the Supplementary Equation 1:

$$\mathrm{TOF}=\frac{\#\text{number of total hydrogen turnovers}/\mathrm{cm}^2_{\mathrm{geo}}}{\#\text{number of active sites}/\mathrm{cm}^2_{\mathrm{geo}}} \quad (1)$$

The total number of hydrogen turnovers was calculated from the current density according to the Supplementary Equation 2:

No. of H_2=

$$\left(j\,\frac{\mathrm{mA}}{\mathrm{cm}^2_{\mathrm{geo}}}\right)\left(\frac{1\ \mathrm{C\ s^{-1}}}{1000\ \mathrm{mA}}\right)\left(\frac{1\ \mathrm{mol\ e^{-1}}}{96485.3\ \mathrm{C}}\right)\left(\frac{1\ \mathrm{mol\ H_2}}{2\ \mathrm{mol\ e^{-}}}\right)\left(\frac{6.022\times10^{23}\ \mathrm{H_{2\ molecules}}}{1\ \mathrm{mol\ H_2}}\right)=3.12\times$$

$$10^{15}\ \frac{\mathrm{H_2\ s^{-}}}{\mathrm{cm}^2_{\mathrm{geo}}}\ \text{per}\ \frac{\mathrm{mA}}{\mathrm{cm}^2_{\mathrm{geo}}} \quad (2)$$

We estimate the number of active sites as the number of surface active oxygen sites (as to be confirmed by the following calculations with H* more easily adsorbing in O than Ru) from the unit cell of the STO, STRO and SRO crystal structure. The active sites per real surface area are calculated from the Supplementary Equations 3:

$$\text{No. of active sites} = \left(\frac{\text{No.of atoms/unit cell}}{\text{No.of atoms/unit cell}}\right)^{\frac{2}{3}} \quad (3)$$

From Figure S10 we can calculate the number of active sites per real surface area for STO, STRO and SRO:

$$\text{No. of active sites (STO)} = \left(\frac{\frac{3}{\text{unit}}\text{cell}}{59.60\frac{\text{Å}^3}{\text{unit}}\text{cell}}\right)^{\frac{2}{3}} = 1.361\times10^{15}\ \text{atoms}\ \mathrm{cm}^{-2}_{\mathrm{real}} \quad (4)$$

$$\text{No. of active sites (STRO)} = \left(\frac{\frac{3}{\text{unit}}\text{cell}}{60.14\frac{\text{Å}^3}{\text{unit}}\text{cell}}\right)^{\frac{2}{3}} = 1.355\times10^{15}\ \text{atoms}\ \mathrm{cm}^{-2}_{\mathrm{real}} \quad (5)$$

$$\text{No. of active sites (SRO)} = \left(\frac{\frac{12}{\text{unit}}\text{cell}}{243.29\frac{\text{Å}^3}{\text{unit}}\text{cell}}\right)^{\frac{2}{3}} = 1.345\times10^{15}\ \text{atoms}\ \mathrm{cm}^{-2}_{\mathrm{real}} \quad (6)$$

The real surface area for HER is calculated from the electrochemical active surface area (ECSA), which can be converted from the specific capacitance. The specific capacitance for a flat surface is generally found to be in the range of 20-60 μF $\mathrm{cm}^2_{\mathrm{geo}}$.

In the following calculations of TOF we assume an average f 40 μF $\mathrm{cm}^2_{\mathrm{geo}}$for STO, STRO and SRO[123, 124].

$$A_{\mathrm{ECSA}}=\frac{\text{specific capacitance}}{40\ \mu\mathrm{F\ cm}^2_{\mathrm{geo}}\ \text{per}\ \mathrm{cm}^2_{\mathrm{ECSA}}} \quad (7)$$

From Figure S8 we can calculate ECSA according to the Supplementary Equation 7 for STO, STRO and SRO:

$$A_{\mathrm{ECSA}}(\mathrm{STO})=\frac{0.31\ \mathrm{mF}/0.196\ \mathrm{cm}^2_{\mathrm{geo}}}{40\ \mu\mathrm{F\ cm}^2_{\mathrm{geo}}\ \text{per}\ \mathrm{cm}^2_{\mathrm{ECSA}}} = 39.5\ \mathrm{cm}^2_{\mathrm{ECSA}} \quad (8)$$

$$A_{\mathrm{ECSA}}(\mathrm{STRO})=\frac{3.26\ \mathrm{mF}/0.196\ \mathrm{cm}^2_{\mathrm{geo}}}{40\ \mu\mathrm{f\ cm}^2_{\mathrm{geo}}\ \text{per}\ \mathrm{cm}^2_{\mathrm{ECSA}}} = 415.8\ \mathrm{cm}^2_{\mathrm{ECSA}} \quad (9)$$

$$A_{ECSA}(SRO)=\frac{1.90\ mF/0.196\ cm^2_{geo}}{40\ \mu F\ cm^2_{geo}\ per\ cm^2_{ECSA}} = 242.3\ cm^2_{ECSA} \quad (10)$$

Finally, the plot of current density can be converted into a TOF plot according to the Supplementary Equation 11:

$$TOF=\frac{3.12\times10^{15}\ \frac{H_2\ s^{-}}{cm^2_{geo}} per\ \frac{mA}{cm^2_{geo}}\times|j|}{No.of\ active\ sites\times A_{ECSA}} \quad (11)$$

Supplementary Note 2. Free energy calculations

Free energies were calculated according to the Supplementary Equation 12:

$$\Delta G_X = \Delta E_X + \Delta ZPE - T\Delta S \quad (12)$$

Where ΔE is the binding energy of the intermediate X ($X = \mathrm{H}^*, \mathrm{H}^* - \mathrm{OH}^*, \mathrm{OH}^*$ and $\mathrm{H_2O}^*$), ΔZPE and $T\Delta S$ are the zero-point energy and entropic contributions at room temperature. The adsorption energies are calculated with respect to H_2 and H_2O, which are taken as reference and their energies are obtained from DFT calculations.

More specifically, the binding energy of H* is obtained according to the Supplementary Equation 13:

$$\Delta E_{\mathrm{H}^*} = E_{\mathrm{H}^*} - E_* - \frac{1}{2}E_{\mathrm{H_2}} \quad (13)$$

Where E_{H^*}, E_* and $E_{\mathrm{H_2}}$ are the energies of the H* intermediate, the bare surface and the energy of a H_2 molecule, respectively. The binding of H*-OH* is obtained via the Supplementary Equation 14:

$$\Delta E_{\mathrm{H}^*-\mathrm{OH}^*} = E_{\mathrm{H}^*-\mathrm{OH}^*} - E_* - E_{\mathrm{H_2O}} \quad (14)$$

Where $E_{\mathrm{H}^*-\mathrm{OH}^*}$ and $E_{\mathrm{H_2O}}$ are the energies of the H*-OH* intermediate (dissociated water molecule) and the energy of a water molecule, respectively.

Finally, the OH* binding energy is calculated according to the Supplementary Equation 15:

$$\Delta E_{\mathrm{H}^*-\mathrm{OH}^*} = E_{\mathrm{OH}^*} - E_* - E_{\mathrm{H_2O}} + \frac{1}{2}E_{\mathrm{H_2}} \quad (15)$$

With E_{OH^*} being the total DFT energy of the OH* intermediate.

The alkaline HER mechanism involves the adsorption and dissociation of H_2O on the surface of the catalyst, followed by the release of OH^- and the subsequent combination of H* with a water molecule (or an adsorbed H*) to release H_2.

Under the Heyrovsky mechanism the overall reaction is:

$$\mathrm{H_2O} + \mathrm{e}^- + * \rightarrow \mathrm{OH}^- + \mathrm{H}^*$$

$$\mathrm{H}^* + \mathrm{H_2O} + \mathrm{e}^- \rightarrow \mathrm{OH}^- + \mathrm{H_2} + *$$

Calculating the free energy of OH^- in solution can be avoided by assuming that the free energies of the above two equations are the same at equilibrium potential of HER. The equation can be broken down into:

$$G_0 = G_* + G_{\mathrm{H_2O}} \quad (16)$$

$$G_1 = G_{\mathrm{H}^*-\mathrm{OH}^*} \quad (17)$$

$$G_2 = G_{\mathrm{H}^*} + G_{\mathrm{OH}^-} \quad (18)$$

$$G_3 = G_* + G_{\mathrm{OH}^-} + \frac{1}{2}G_{\mathrm{H_2}} \quad (19)$$

With $G_0 = G_3$. Hence the reaction energy profile can be constructed by considering the free energy changes using the total free energies defined above. This gives: $\Delta G_{H^*-OH^*} = G_1 - G_0$ for the Volmer step, while the activity of the Heyrovsky step is described using $\Delta G_{H^*} = G_2 - G_3$.

Supplementary References:

1. McCrory, C. C. L., Jung, S., Peters, J. C. & Jaramillo, T. F. Benchmarking heterogeneous electrocatalysts for the oxygen evolution reaction. *J. Am. Chem. Soc.* **135**, 16977-16987 (2013).
2. Yin, Y. *et al.* Synergistic phase and disorder engineering in 1T-$MoSe_2$ nanosheets for enhanced hydrogen-evolution reaction. *Adv. Mater.* **29**, 1700311 (2017).
3. Zhu, Y. *et al.* A perovskite nanorod as bifunctional electrocatalyst for overall water splitting. *Adv. Energy Mater.* **7**, 1602122 (2017).
4. Zhu, Y. *et al.* Unusual synergistic effect in layered Ruddlesden−Popper oxide enables ultrafast hydrogen evolution. *Nat. Commun.* **10**, 149 (2019).
5. Guan, D. *et al.* Screening highly active perovskites for hydrogen-evolving reaction via unifying ionic electronegativity descriptor. *Nat. Commun.* **10**, 3755 (2019).
6. Ling, T. *et al.* Activating cobalt(II) oxide nanorods for efficient electrocatalysis by strain engineering. *Nat. Commun.* **8**, 1509 (2017).
7. Wang, H. *et al.* Bifunctional non-noble metal oxide nanoparticle electrocatalysts through lithium-induced conversion for overall water splitting. *Nat. Commun.* **6**, 7261 (2015).
8. Hua, B. *et al.* Activating p-Blocking Centers in Perovskite for Efficient Water Splitting. *Chem* **4**, 2902-2916 (2018).
9. Peng, S. *et al.* Necklace-like multishelled hollow spinel oxides with oxygen vacancies for efficient water electrolysis. *J. Am. Chem. Soc.* **140**, 13644-13653 (2018).
10. Zhu, Y. P., Ma, T. Y., Jaroniec, M. & Qiao, S. Z. Self-templating synthesis of hollow Co_3O_4 microtube arrays for highly efficient water electrolysis. *Angew. Chem. Int. Ed.* **56**, 1324-1328 (2017).
11. Gao, X. *et al.* Hierarchical $NiCo_2O_4$ hollow microcuboids as bifunctional electrocatalysts for overall water-splitting. *Angew. Chem. Int. Ed.* **55**, 6290-6294 (2016).
12. Huang, L. *et al.* Zirconium-regulation-induced bifunctionality in 3D cobalt–iron oxide nanosheets for overall water splitting. *Adv. Mater.* **31**, 1901439 (2019).
13. Ling, T. *et al.* Well-dispersed nickel- and zinc-tailored electronic structure of a transition metal oxide for highly active alkaline hydrogen evolution reaction. *Adv. Mater.* **31**, 1807771 (2019).
14. Chen, G. *et al.* A universal strategy to design superior water-splitting electrocatalysts based on fast in situ reconstruction of amorphous nanofilm precursors. *Adv. Mater.* **30**, 1804333 (2018).
15. Xu, X. *et al.* A perovskite electrocatalyst for efficient hydrogen evolution reaction. *Adv. Mater.* **28**, 6442-6448 (2016).
16. Xiao, Z. *et al.* Filling the oxygen vacancies in Co_3O_4 with phosphorus: an ultra-efficient electrocatalyst for overall water splitting. *Energy Environ. Sci.* **10**, 2563-2569 (2017).
17. Kuang, M., Han, P., Wang, Q., Li, J. & Zheng, G. CuCo hybrid oxides as bifunctional electrocatalyst for efficient water splitting. *Adv. Funct. Mater.* **26**, 8555-8561 (2016).

18. Zhang, T. *et al.* Engineering oxygen vacancy on NiO nanorod arrays for alkaline hydrogen evolution. *Nano Energy* **43**, 103-109 (2018).
19. Cobo, S. *et al.* A Janus cobalt-based catalytic material for electro-splitting of water. *Nat. Mater.* **11**, 802-807 (2012).
20. Oh, N. K. *et al.* In-situ local phase-transitioned $MoSe_2$ in $La_{0.5}Sr_{0.5}CoO_{3-\delta}$ heterostructure and stable overall water electrolysis over 1000 hours. *Nat. Commun.* **10**, 1723 (2019).
21. Huang, Y. *et al.* Atomically engineering activation sites onto metallic 1T-MoS_2 catalysts for enhanced electrochemical hydrogen evolution. *Nat. Commun.* **10**, 982 (2019).
22. Wang, P. *et al.* Precise tuning in platinum-nickel/nickel sulfide interface nanowires for synergistic hydrogen evolution catalysis. *Nat. Commun.* **8**, 14580 (2017).
23. Yin, H. *et al.* Ultrathin platinum nanowires grown on single-layered nickel hydroxide with high hydrogen evolution activity. *Nat. Commun.* **6**, 6430 (2015).
24. Gong, M. *et al.* Nanoscale nickel oxide/nickel heterostructures for active hydrogen evolution electrocatalysis. *Nat. Commun.* **5**, 4695 (2014).
25. Hu, J. *et al.* Nanohybridization of MoS_2 with layered double hydroxides efficiently synergizes the hydrogen evolution in alkaline media. *Joule* **1**, 383-393 (2017).
26. Lu, K. *et al.* Li_xNiO/Ni heterostructure with strong basic lattice oxygen enables electrocatalytic hydrogen evolution with Pt-like activity. *J. Am. Chem. Soc.* **142**, 12613-12619 (2020).
27. Zhao, Z. *et al.* Surface-engineered PtNi-O nanostructure with record-high performance for electrocatalytic hydrogen evolution reaction. *J. Am. Chem. Soc.* **140**, 9046-9050 (2018).
28. Zhao, D. *et al.* Synergistically interactive pyridinic-N–MoP sites: identified active centers for enhanced hydrogen evolution in alkaline solution. *Angew. Chem. Int. Ed.* **59**, 8982-8990 (2020).
29. You, B., Zhang, Y., Jiao, Y., Davey, K. & Qiao, S. Z. Negative charging of transition-metal phosphides via strong electronic coupling for destabilization of alkaline water. *Angew. Chem. Int. Ed.* **58**, 11796-11800 (2019).
30. Li, F. *et al.* Construction of porous Mo_3P/Mo nanobelts as catalysts for efficient water splitting. *Angew. Chem. Int. Ed.* **57**, 14139-14143 (2018).
31. Feng, J. X., Xu, H. Dong, Y. T., Lu, X. F., Tong, Y. X. & Li, G. R. Efficient hydrogen evolution electrocatalysis using cobalt nanotubes decorated with titanium dioxide nanodots. *Angew. Chem. Int. Ed.* **56**, 2960-2964 (2017).
32. He, Q. *et al.* Achieving efficient alkaline hydrogen evolution reaction over a Ni_5P_4 catalyst incorporating single-atomic Ru sites. *Adv. Mater.* **32**, 1906972 (2020).
33. Zhu Z, *et al.* Ultrathin transition metal dichalcogenide/3d metal hydroxide hybridized nanosheets to enhance hydrogen evolution activity. *Adv. Mater.* **30**, 1801171 (2018).
34. Hou, Y., Lohe, M. R., Zhang, J., Liu, S., Zhuang, X. & Feng, X. Vertically oriented cobalt selenide/NiFe layered-double-hydroxide nanosheets supported on exfoliated graphene foil: an efficient 3D electrode for overall water splitting. *Energy Environ. Sci.* **9**, 478-483 (2016).

35. Yan, X., Tian, L., He, M. & Chen, X. Three-dimensional crystalline/amorphous Co/Co_3O_4 core/shell nanosheets as efficient electrocatalysts for the hydrogen evolution reaction. *Nano Lett.* **15**, 6015-6021 (2015).
36. Wang, D., Li, Q., Han, C., Xing, Z. & Yang, X. When NiO@Ni meets WS_2 nanosheet array: a highly efficient and ultrastable electrocatalyst for overall water splitting. *ACS Cent. Sci.* **4**, 112-119 (2018).
37. Wang, Y., Chen, L., Yu, X., Wang, Y. & Zheng, G. Superb alkaline hydrogen evolution and simultaneous electricity generation by Pt-decorated Ni_3N nanosheets. *Adv. Energy Mater.* **7**, 1601390 (2017).
38. Ibupoto, Z. H. *et al.* MoS_x@NiO Composite nanostructures: an advanced nonprecious catalyst for hydrogen evolution reaction in alkaline media. *Adv. Funct. Mater.* **29**, 1807562 (2019).
39. Wang, Y., Ma, J., Wang, J., Chen, S., Wang, H. & Zhang, J. Interfacial scaffolding preparation of hierarchical PBA-based derivative electrocatalysts for efficient water splitting. *Adv. Energy Mater.* **9**, 1802939 (2019).
40. Xiao, C., Li, Y., Lu, X. & Zhao, C. Bifunctional porous $NiFe/NiCo_2O_4/Ni$ foam electrodes with triple hierarchy and double synergies for efficient whole cell water splitting. *Adv. Funct. Mater.* **26**, 3515-3523 (2016).
41. An, L., Huang, L., Zhou, P., Yin, J., Liu, H & Xi, P. A self-standing high-performance hydrogen evolution electrode with nanostructured $NiCo_2O_4/CuS$ heterostructures. *Adv. Funct. Mater.* **25**, 6814-6822 (2015).
42. Shao, Q. *et al.* Stabilizing and activating metastable nickel nanocrystals for highly efficient hydrogen evolution electrocatalysis. *ACS Nano* **12**, 11625-11631 (2018).
43. Luo, Y. *et al.* Two-dimensional MoS_2 confined $Co(OH)_2$ electrocatalysts for hydrogen evolution in alkaline electrolytes. *ACS Nano* **12**, 4565-4573 (2018).
44. Yu, X. *et al.* Hydrogen evolution reaction in alkaline media: alpha- or beta-nickel hydroxide on the surface of platinum? *ACS Energy Lett.* **3**, 237-244 (2018).
45. Chen, Y. *et al.* Electrocatalytically inactive SnS_2 promotes water adsorption/dissociation on molybdenum dichalcogenides for accelerated alkaline hydrogen evolution. *Nano Energy* **64**, 103918 (2019).
46. Liu, S. Q. *et al.* Amorphous $Ni(OH)_2$ encounter with crystalline CuS in hollow spheres: A mesoporous nano-shelled heterostructure for hydrogen evolution electrocatalysis. *Nano Energy* **44**, 7-14 (2018).
47. Zhang, B. *et al.* Interface engineering: The $Ni(OH)_2/MoS_2$ heterostructure for highly efficient alkaline hydrogen evolution. *Nano Energy* **37**, 74-80 (2017).
48. Xing, Z., Han, C., Wang, D., Li, Q. & Yang, X. Ultrafine Pt nanoparticle-decorated $Co(OH)_2$ nanosheet arrays with enhanced catalytic activity toward hydrogen evolution. *ACS Catal.* **7**, 7131-7135 (2017).
49. Lu, Q. *et al.* Crystal phase-based epitaxial growth of hybrid noble metal nanostructures on 4H/fcc Au nanowires. *Nat. Chem.* **10**, 456-461 (2018).
50. Li, Y., Wei, X., Chen, L., Shi, J. & He, M. Nickel-molybdenum nitride nanoplate electrocatalysts for concurrent electrolytic hydrogen and formate productions. *Nat. Commun.* **10**, 5335 (2019).

51. Hu, C. *et al.* In situ electrochemical production of ultrathin nickel nanosheets for hydrogen evolution electrocatalysis. *Chem* **3**, 122-133 (2017).
52. Lao, M. *et al.* Platinum/nickel bicarbonate heterostructures towards accelerated hydrogen evolution under alkaline conditions. *Angew. Chem. Int. Ed.* **58**, 5432-5437 (2019).
53. Menezes, P. W., Panda, C., Garai, S., Walter, C., Guiet, A. & Driess, M. Structurally ordered intermetallic cobalt stannide nanocrystals for high-performance electrocatalytic overall water-splitting. *Angew. Chem. Int. Ed.* **57**, 15237-15242 (2018).
54. Wang, P., Jiang, K., Wang, G., Yao, J. & Huang, X. Phase and interface engineering of platinum–nickel nanowires for efficient electrochemical hydrogen evolution. *Angew. Chem. Int. Ed.* **55**, 12859-12863 (2016).
55. Shen, Y., Zhou, Y., Wang, D., Wu, X., Li, J. & Xi, J. Nickel–copper alloy encapsulated in graphitic carbon shells as electrocatalysts for hydrogen evolution reaction. *Adv. Energy Mater.* **8**, 1701759 (2018).
56. Li, T. *et al.* Encapsulation of Ni_3Fe nanoparticles in N-doped carbon nanotube–grafted carbon nanofibers as high-efficiency hydrogen evolution electrocatalysts. *Adv. Funct. Mater.* **28**, 1805828 (2018).
57. Sun, J. S., Wen, Z., Han, L. P., Chen, Z. W., Lang, X. Y. & Jiang Q. Nonprecious intermetallic Al_7Cu_4Ni nanocrystals seamlessly integrated in freestanding bimodal nanoporous copper for efficient hydrogen evolution catalysis. *Adv. Funct. Mater.* **28**, 1706127 (2018).
58. Schäfer, H. *et al.* Electro-oxidation of Ni42 steel: a highly active bifunctional electrocatalyst. *Adv. Funct. Mater.* **26**, 6402-6417 (2016).
59. Zeng, M. *et al.* Metallic cobalt nanoparticles encapsulated in nitrogen-enriched graphene shells: its bifunctional electrocatalysis and application in Zinc–air batteries. *Adv. Funct. Mater.* **26**, 4397-4404 (2016).
60. Ledendecker, M., Clavel, G., Antonietti, M. & Shalom, M. Highly porous materials as tunable electrocatalysts for the hydrogen and oxygen evolution reaction. *Adv. Funct. Mater.* **25**, 393-399 (2015).
61. Fang, M. *et al.* Hierarchical NiMo-based 3D electrocatalysts for highly-efficient hydrogen evolution in alkaline conditions. *Nano Energy* **27**, 247-254 (2016).
62. Li, Z. *et al.* Mesoporous hollow Cu–Ni alloy nanocage from core–shell Cu@Ni nanocube for efficient hydrogen evolution reaction. *ACS Catal.* **9**, 5084-5095 (2019).
63. Cao, L. *et al.* Identification of single-atom active sites in carbon-based cobalt catalysts during electrocatalytic hydrogen evolution. *Nat. Catal.* **2**, 134-141 (2019).
64. Zhang, H. *et al.* Dynamic traction of lattice-confined platinum atoms into mesoporous carbon matrix for hydrogen evolution reaction. *Sci. Adv.* **4**, eaao6657 (2018).
65. Fang, S. *et al.* Uncovering near-free platinum single-atom dynamics during electrochemical hydrogen evolution reaction. *Nat. Commun.* **11**, 1029 (2020).

66. Jiang, K. *et al.* Single platinum atoms embedded in nanoporous cobalt selenide as electrocatalyst for accelerating hydrogen evolution reaction. *Nat. Commun.* **10**, 1743 (2019).
67. Fei, H. *et al.* Atomic cobalt on nitrogen-doped graphene for hydrogen generation. *Nat. Commun.* **6**, 8668 (2015).
68. Liang, H. W., Brüller, S. Dong, R., Zhang, J. Feng, X. & Müllen, K. Molecular metal–N_x centres in porous carbon for electrocatalytic hydrogen evolution. *Nat. Commun.* **6**, 7992 (2015).
69. Chen, W. *et al.* Rational design of single molybdenum atoms anchored on N-doped carbon for effective hydrogen evolution reaction. *Angew. Chem. Int. Ed.* **56**, 16086-16090 (2017).
70. Fang, Z. *et al.* Dual tuning of Ni–Co–A (A = P, Se, O) nanosheets by anion substitution and holey engineering for efficient hydrogen evolution. *J. Am. Chem. Soc.* **140**, 5241-5247 (2018).
71. Pan, Y. *et al.* Core–shell ZIF-8@ZIF-67-derived CoP nanoparticle-embedded N-doped carbon nanotube hollow polyhedron for efficient overall water splitting. *J. Am. Chem. Soc.* **140**, 2610-2618 (2018).
72. Tian, J., Liu, Q., Asiri, A. M. & Sun, X. Self-supported nanoporous cobalt phosphide nanowire arrays: an efficient 3D hydrogen-evolving cathode over the wide range of pH 0–14. *J. Am. Chem. Soc.* **136**, 7587-7590 (2014).
73. Pu, Z., Amiinu, I. S., Kou, Z., Li, W. & Mu, S. RuP_2-based catalysts with platinum-like activity and higher durability for the hydrogen evolution reaction at all pH values. *Angew. Chem. Int. Ed.* **56**, 11559-11564 (2017).
74. Ledendecker, M., Krick, Calderón. S., Papp, C., Steinrück, H. P., Antonietti, M. & Shalom, M. The synthesis of nanostructured Ni_5P_4 films and their use as a non-noble bifunctional electrocatalyst for full water splitting. *Angew. Chem. Int. Ed.* **54**, 12361-12365 (2015).
75. Tang, C. *et al.* Fe-doped CoP nanoarray: a monolithic multifunctional catalyst for highly efficient hydrogen generation. *Adv. Mater.* **29**, 1602441 (2017).
76. Ma, Y. Y. *et al.* Highly efficient hydrogen evolution from seawater by a low-cost and stable CoMoP@C electrocatalyst superior to Pt/C. *Energy Environ. Sci.* **10**, 788-798 (2017).
77. Tan, Y. *et al.* Versatile nanoporous bimetallic phosphides towards electrochemical water splitting. *Energy Environ. Sci.* **9**, 2257-2261 (2016).
78. Xiao, P. *et al.* Molybdenum phosphide as an efficient electrocatalyst for the hydrogen evolution reaction. *Energy Environ. Sci.* **7**, 2624-2629 (2014).
79. Li, G. *et al.* Carbon-tailored semimetal MoP as an efficient hydrogen evolution electrocatalyst in both alkaline and acid media. *Adv. Energy Mater.* **8**, 1801258 (2018).
80. Liu, T. *et al.* Enhanced electrocatalysis for energy-efficient hydrogen production over CoP catalyst with nonelectroactive Zn as a promoter. *Adv. Energy Mater.* **7**, 1700020 (2017).

81. Song, J. *et al.* Bimetallic cobalt-based phosphide zeolitic imidazolate framework: CoP_x phase-dependent electrical conductivity and hydrogen atom adsorption energy for efficient overall water splitting. *Adv. Energy Mater.* **7**, 1601555 (2017).
82. Zhang, X., Yu, X., Zhang, L., Zhou, F., Liang, Y. & Wang, R. Molybdenum phosphide/carbon nanotube hybrids as pH-universal electrocatalysts for hydrogen evolution reaction. *Adv. Funct. Mater.* **28**, 1706523 (2018).
83. Li, J. *et al.* Mechanistic insights on ternary $Ni_{2-x}Co_xP$ for hydrogen evolution and their hybrids with graphene as highly efficient and robust catalysts for overall water splitting. *Adv. Funct. Mater.* **26**, 6785-6796 (2016).
84. Wang, X., Li, W., Xiong, D., Petrovykh, D. Y. & Liu, L. Bifunctional nickel phosphide nanocatalysts supported on carbon fiber paper for highly efficient and stable overall water splitting. *Adv. Funct. Mater.* **26**, 4067-4077 (2016).
85. Zhu, Y. P., Liu, Y. P., Ren, T. Z. & Yuan, Z. Y. Self-supported cobalt phosphide mesoporous nanorod arrays: a flexible and bifunctional electrode for highly active electrocatalytic water reduction and oxidation. *Adv. Funct. Mater.* **25**, 7337-7347 (2015).
86. Gao, W. *et al.* Modulating electronic structure of CoP electrocatalysts towards enhanced hydrogen evolution by Ce chemical doping in both acidic and basic media. *Nano Energy* **38**, 290-296 (2017).
87. Yu, L. *et al.* In situ growth of Ni_2P–Cu_3P bimetallic phosphide with bicontinuous structure on self-supported NiCuC substrate as an efficient hydrogen evolution reaction electrocatalyst. *ACS Catal.* **9**, 6919-6928 (2019).
88. Zang, Y. *et al.* Tuning orbital orientation endows molybdenum disulfide with exceptional alkaline hydrogen evolution capability. *Nat. Commun.* **10**, 1217 (2019).
89. Feng, J. X., Wu, J. Q., Tong, Y. X. & Li, G. R. Efficient hydrogen evolution on Cu nanodots-decorated Ni_3S_2 nanotubes by optimizing atomic hydrogen adsorption and desorption. *J. Am. Chem. Soc.* **140**, 610-617 (2018).
90. Huang, Z. F. *et al.* Hollow cobalt-based bimetallic sulfide polyhedra for efficient all-pH-value electrochemical and photocatalytic hydrogen evolution. *J. Am. Chem. Soc.* **138**, 1359-1365 (2016).
91. Feng, L. L. *et al.* High-index faceted Ni_3S_2 nanosheet arrays as highly active and ultrastable electrocatalysts for water splitting. *J. Am. Chem. Soc.* **137**, 14023-14026 (2015).
92. Zhang, J. *et al.* Interface engineering of MoS_2/Ni_3S_2 heterostructures for highly enhanced electrochemical overall-water-splitting Activity. *Angew. Chem. Int. Ed.* **55**, 6702-6707 (2016).
93. Zhang, J. *et al.* Engineering water dissociation sites in MoS_2 nanosheets for accelerated electrocatalytic hydrogen production. *Energy Environ. Sci.* **9**, 2789-2793 (2016).
94. Kou, T. *et al.* Theoretical and experimental insight into the effect of nitrogen doping on hydrogen evolution activity of Ni_3S_2 in alkaline medium. *Adv. Energy Mater.* **8**, 1703538 (2018).

95. Peng, Z., Jia, D., Al-Enizi, A. M., Elzatahry, A. A. & Zheng, G. From water oxidation to reduction: homologous Ni–Co based nanowires as complementary water splitting electrocatalysts. *Adv. Energy Mater.* **5**, 1402031 (2015).
96. Wang, J. *et al.* Activating basal planes of $NiPS_3$ for hydrogen evolution by nonmetal heteroatom doping. *Adv. Funct. Mater.* **30**, 1908708 (2020).
97. Kang, Z. *et al.* Engineering an earth-abundant element-based bifunctional electrocatalyst for highly efficient and durable overall water splitting. *Adv. Funct. Mater.* **29**, 1807031 (2019).
98. Sivanantham, A., Ganesan, P. & Shanmugam, S. Hierarchical $NiCo_2S_4$ Nanowire arrays supported on Ni foam: an efficient and durable bifunctional electrocatalyst for oxygen and hydrogen evolution reactions. *Adv. Funct. Mater.* **26**, 4661-4672 (2016).
99. Cao, D. *et al.* Engineering the in-plane structure of metallic phase molybdenum disulfide via Co and O dopants toward efficient alkaline hydrogen evolution. *ACS Nano* **13**, 11733-11740 (2019).
100. Liu, H. *et al.* Electronic structure reconfiguration toward pyrite NiS_2 via engineered heteroatom defect boosting overall water splitting. *ACS Nano* **11**, 11574-11583 (2017).
101. Yang, C., Gao, M. Y., Zhang, Q. B., Zeng, J. R., Li, X. T. & Abbott, A. P. In-situ activation of self-supported 3D hierarchically porous Ni_3S_2 films grown on nanoporous copper as excellent pH-universal electrocatalysts for hydrogen evolution reaction. *Nano Energy* **36**, 85-94 (2017).
102. Zheng, Y. R. *et al.* Doping-induced structural phase transition in cobalt diselenide enables enhanced hydrogen evolution catalysis. *Nat. Commun.* **9**, 2533 (2018).
103. Zheng, T. *et al.* Intercalated iridium diselenide electrocatalysts for efficient pH-universal water splitting. *Angew. Chem. Int. Ed.* **58**, 14764-14769 (2019).
104. Zhang, J. Y. *et al.* Anodic hydrazine oxidation assists energy-efficient hydrogen evolution over a bifunctional cobalt perselenide nanosheet electrode. *Angew. Chem. Int. Ed.* **57**, 7649-7653 (2018).
105. Tang, C., Cheng, N., Pu, Z., Xing, W. & Sun, X. NiSe nanowire film supported on nickel foam: an efficient and stable 3D bifunctional electrode for full water splitting. *Angew. Chem. Int. Ed.* **54**, 9351-9355 (2015).
106. Liu, B. *et al.* Nickel–cobalt diselenide 3D mesoporous nanosheet networks supported on Ni foam: an all-pH highly efficient integrated electrocatalyst for hydrogen evolution. *Adv. Mater.* **29**, 1606521 (2017).
107. Chen, P. *et al.* Phase-transformation engineering in cobalt diselenide realizing enhanced catalytic activity for hydrogen evolution in an alkaline medium. *Adv. Mater.* **28**, 7527-7532 (2016).
108. Najafi, L. *et al.* Engineered $MoSe_2$-based heterostructures for efficient electrochemical hydrogen evolution reaction. *Adv. Energy Mater.* **8**, 1703212 (2018).

109. Wu, H. B., Xia, B. Y., Yu, L., Yu, X. Y. & Lou, X. W. Porous molybdenum carbide nano-octahedrons synthesized via confined carburization in metal-organic frameworks for efficient hydrogen production. *Nat. Commun.* **6**, 6512 (2015).
110. Vrubel, H. & Hu, X. Molybdenum boride and carbide catalyze hydrogen evolution in both acidic and basic solutions. *Angew. Chem. Int. Ed.* **51**, 12703-12706 (2012).
111. Li, S., Yang, J., Song, C., Zhu, Q., Xiao, D. & Ma, D. Iron carbides: control synthesis and catalytic applications in CO_x hydrogenation and electrochemical HER. *Adv. Mater.* **31**, 1901796 (2019).
112. Huang, Y., Ge, J., Hu, J., Zhang, J., Hao, J. & Wei, Y. Nitrogen-doped porous molybdenum carbide and phosphide hybrids on a carbon matrix as highly effective electrocatalysts for the hydrogen evolution reaction. *Adv. Energy Mater.* **8**, 1701601 (2018).
113. Huang, Y. *et al.* Mo_2C nanoparticles dispersed on hierarchical carbon microflowers for efficient electrocatalytic hydrogen evolution. *ACS Nano* **10**, 11337-11343 (2016).
114. Chen, Y. Y. *et al.* Pomegranate-like N,P-doped Mo_2C@C nanospheres as highly active electrocatalysts for alkaline hydrogen evolution. *ACS Nano* **10**, 8851-8860 (2016).
115. Jia, J. *et al.* Molybdenum carbide on hierarchical porous carbon synthesized from Cu-MoO_2 as efficient electrocatalysts for electrochemical hydrogen generation. *Nano Energy* **41**, 749-757 (2017).
116. Lai, J., Huang, B., Chao, Y., Chen, X. & Guo, S. Strongly coupled nickel–cobalt nitrides/carbon hybrid nanocages with Pt-like activity for hydrogen evolution catalysis. *Adv. Mater.* **31**, 1805541 (2019).
117. Zhu, C. *et al.* In situ grown epitaxial heterojunction exhibits high-performance electrocatalytic water splitting. *Adv. Mater.* **30**, 1705516 (2018).
118. Zhang, Y., Ouyang, B., Xu, J., Chen, S., Rawat, R. S. & Fan, H. J. 3D porous hierarchical nickel–molybdenum nitrides synthesized by RF plasma as highly active and stable hydrogen-evolution-reaction electrocatalysts. *Adv. Energy Mater.* **6**, 1600221 (2016).
119. Zhou, M. *et al.* Construction of polarized carbon–nickel catalytic surfaces for potent, durable, and economic hydrogen evolution reactions. *ACS Nano* **12**, 4148-4155 (2018).
120. Zheng, Y. *et al.* Hydrogen evolution by a metal-free electrocatalyst. *Nat. Commun.* **5**, 3783 (2014).
121. Lai, J., Li, S., Wu, F., Saqib, M., Luque, R. & Xu, G. Unprecedented metal-free 3D porous carbonaceous electrodes for full water splitting. *Energy Environ. Sci.* **9**, 1210-1214 (2016).
122. Qu, K., Zheng, Y., Zhang, X., Davey, K., Dai, S. & Qiao, S. Z. Promotion of electrocatalytic hydrogen evolution reaction on nitrogen-doped carbon nanosheets with secondary heteroatoms. *ACS Nano* **11**, 7293-7300 (2017).

123. Kibsgaard, J. & Jaramillo, T. F. Molybdenum phosphosulfide: Aan active, acid-stable, earth-abundant catalyst for the hydrogen evolution reaction. *Angew. Chem. Int. Ed.* **53**, 14433-14437 (2014).
124. Kibsgaard, J. *et al.* Designing an improved transition metal phosphide catalyst for hydrogen evolution using experimental and theoretical trends. *Energy Environ. Sci.* **8**, 3022-3029 (2015).
